# Disentangling Physical Activity and Sedentary Behavior Patterns in Children with Low Motor Competence

**DOI:** 10.3390/ijerph16203804

**Published:** 2019-10-10

**Authors:** Dave H.H. Van Kann, Anoek M. Adank, Martin L. van Dijk, Teun Remmers, Steven B. Vos

**Affiliations:** 1 School of Sport Studies, Fontys University of Applied Sciences, P.O. Box 347, 5600 AH Eindhoven, The Netherlands; a.adank@fontys.nl (A.M.A.); martin.vandijk@fontys.nl (M.L.v.D.); t.remmers@fontys.nl (T.R.); s.vos@tue.nl (S.B.V.); 2 Department of Health Promotion, Nutrition and Translational Research Institute Maastricht (NUTRIM), Maastricht University, P.O. Box 616, 6200 MD Maastricht, The Netherlands; 3 Department of Industrial Design, Eindhoven University of Technology, 5600 MB Eindhoven, The Netherlands

**Keywords:** low motor competence, physical activity, sedentary behavior, primary schools, children

## Abstract

Children with low motor competence (MC) are at high-risk for physical inactivity, yet little is known about their physical activity (PA) and sedentary behavior (SB) patterns throughout the day. The purpose of this study is to disentangle PA and SB patterns among children with low MC across segmented day periods taking into account differences in gender and age. Data collection took place between May and July 2017. The Athletic Skills Track was used to measure MC. PA levels were objectively measured using accelerometers (ActiGraph, GT3X+) on school days. Data were segmented for (1) time before school, (2) time during school (based on school schedules), and (3) time after school. In total, data from 117 7-to-11 years-old children with low MC were eligible for analyses (*N* = 58 girls; *N* = 59 boys). Differences in moderate-to-vigorous PA (MVPA) and SB between segmented periods, gender, and grade were analyzed by ANOVAs with post hoc tests (Tukey) and Independent Sample T-tests respectively. Time spent at school is the major contributor of time spent in SB in children with low MC. Low MC is equally distributed among gender, but large differences exist among boys and girls in both MVPA and SB, indicating low-MC girls as most inactive group. This pattern is found in all segmented periods of the school day, i.e., before, during, and after school. This study stresses the negative contribution of current school curricula on PA and SB in children with low MC, indicating the most efficient period of the day to intervene. Future school-based PA and SB interventions should particularly focus on specific high-risk populations, i.e., children with low MC, and girls in particular.

## 1. Introduction

Many young people’s physical activity (PA) levels are insufficient to gain health benefits [1,2]. Besides insufficient PA and excessive sedentary behavior (SB), declines in elements of motor competence (MC), such as motor fitness, have been reported over the last decades [3]. The term MC is an umbrella concept to describe goal-directed human behavior and is covering a wide variety of terminology (e.g., fundamental movement skills and motor ability) [4]. Low MC has a negative impact on the physical, cognitive, and social development of children [5]. Several studies in children showed that MC is related to PA levels [6,7,8] and sedentary behavior (SB) [9,10]. Children with poor MC spend less time in moderate-to-vigorous physical activity (MVPA) compared to other children with normal MC [11]. In addition, Adank et al. [12] and Lopes et al. [9] found that children with low MC spend more time sedentary compared to their counterparts. Moreover, a reciprocal relationship is assumed between MC and PA levels [3,13,14,15], indicating that motor skills development might be stimulated by increasing PA levels or decreasing SB. Yet, as a result of the reciprocal association between PA and MC, children with low MC are more at risk for developing and maintaining an inactive lifestyle [15,16,17]. 

Current efforts to increase children’s PA levels and reduce SB resulted in small and short term effects [18]. Many PA supporting activities are implemented as a generic program, insufficiently taking into account different needs of different target populations. This is particularly the case in school-based PA programs, which might be debit on the lack of effectiveness of such programs [19], whereas its potential in the school environment is present [20]. Generic programs tend to follow a one-size-fits-all approach, whereas socio-ecological frameworks, such as the Environmental Research framework for weight Gain prevention (EnRG) framework [21], define rather stable individual characteristics (e.g., gender and age) as moderators of the relationship between environmental exposure and PA behavior. According to this framework, MC can be seen as a moderator and different approaches might be needed to affect children’s PA and SB with low MC compared to children with normal and high MC. The need to differentiate is also seen in well-studied demographic variables, such as age and gender. In general, boys tend to be more active and less sedentary compared to girls [14,22,23], whereas daily levels of PA tend to increase up to an age of approximately 10–11 years old and decrease afterwards heading into puberty [23,24,25].

To develop effective PA interventions for children, particularly for children at high-risk for inactivity (i.e., children with low MC), it is important to get more insight into their PA and SB patterns and related factors. In addition, the impact of determinants that influence SB or PA depends on the context in which these behaviors occur [26]. Therefore, it is warranted to investigate more meaningful relationships between contextually matched determinants and segregated PA and SB patterns [27]. A first important context that may affect how determinants influence PA and SB patterns is time period of the day [28]. A few studies investigated segmented PA levels during school days [29,30,31]. Other studies investigated the contribution of specific time intervals in the total amount of PA, such as time before school [32], time at school, and time after school [33,34,35]. Moreover, Remmers and colleagues [28] found that within these different time periods, the relationship between environmental exposure and PA levels differed, stressing the urge to clearly explore the opportunities to optimize timing of PA interventions. 

Improved insight into PA and SB patterns of children with low MC at distinct and meaningful periods of the school day might provide useful contextual information regarding the potential time frames in which PA interventions for these children could be implemented best. Therefore, the current study’s focus is on disentangling daily PA and SB patterns (i.e., before school, during school, and after school) to identify potential periods of time to effectively affect PA and SB in children with low MC.

## 2. Materials and Methods 

### 2.1. Study Sample and Procedure

Data used in the current study derived from the SALTO (Dutch acronym for Stimulating an Active Lifestyle Through Physical Education) study [12]. This study was conducted on ten primary schools in the south of the Netherlands. A total of 1125 grade-4 and grade-6 children (aged 7–11 years old) participated in this study. In the Netherlands primary schools range from grade 1 till grade 8. Data collection took place between May and July 2017.

In participating schools, all grade-4 and grade-6-children were informed about the SALTO study. Further, their parents/caretakers were informed about the content and goals of the study and asked to provide written informed consent to measure children’s anthropometry and objectively assess their PA levels through accelerometry. MC assessment was part of a regular physical education (PE) lesson. Ethical approval for the SALTO study was obtained by the Ethical Research Committee of Fontys University of Applied Sciences (reference number FCEO 24-03 Adank).

### 2.2. Instruments/Measures

The Athletic Skills Track (AST) was used to measure MC. This test has been shown to be a reliable and valid tool to assess MC in elementary school children [36]. The test consisted of a series of fundamental movement tasks, e.g., locomotive and stability skills, to be completed as fast as possible and age-adjustments were applied in this circuit-based assessment. Grade-4 children completed the AST-2 circuit consisting of (1) walking, (2) traveling jumps, (3) hopscotch, (4) alligator crawl (backwards), (5) running (backwards), (6) pencil roll, and (7) clambering, whereas grade-6 children completed the AST-3 circuit consisting of (1) walking (backwards), (2) traveling jumps, (3) hopscotch (backwards), (4) alligator crawl (backwards), (5) slaloming (backwards), (6) forward roll, and (7) clambering [36]. Based on gender- and age-specific cut-off values [37], children were classified as (very) low skilled (i.e., scores 1 and 2), averagely skilled (score 3) and (very) high skilled (score 4 and 5). Children scoring 1 or 2 were eligible for the purpose of the current study (*N* = 189).

PA and SB were objectively measured using ActiGraph GT3X+ accelerometers (ActiGraph, Pensacola, FL, USA). As part of the SALTO protocol, children were instructed to wear the accelerometer for seven consecutive days during waking hours, only removing the device while performing water-involved activities, such as swimming and showering. The accelerometer was attached to an elastic belt and children wore the device on their right hip. For the purpose of the current study, only weekdays were included to ensure a certain level of homogeneity throughout daily activity patterns and definition of meaningful patterns [38]. A minimum of 8 h of valid wear time during waking hours (defined as 0600–2300) was needed to be included in the analyses and at least three valid school days were required. Wear time validation was calculated using Choi’s criteria [39]. MVPA and SB were determined by Evenson’s cut-off values, i.e., SB < 100 counts-per-minute (CPM) and MVPA > 2295 CPM [40]. MVPA and SB levels before school were assessed between 0600 (getting up) and the start of the school day (i.e., 0830 or 0845, depending each school’s specific schedule). Time before school data were included when at least 30 min of accelerometer data in this time segment was available. As no clear criteria are defined yet, we applied a conservative approach on the wear time validation criterion in this time period. Thirty minutes was assumed to be the minimum children need to get up, take on their clothes, and travel to school. Time during school was included in the analyses when available accelerometer wear time was equal to the time spent at school (which was the case in >99% of the study population). Time after school was defined as end of school operating hours (varying between 1415 and 1530) up to 2300. To be included in analyses, at least 50% of valid wear time data in this time segment was required [28]. The distinction in three meaningful periods was based on equal time spent in one certain location, i.e., school. All three periods contained other PA and SB opportunities: ‘time before school’ mostly consisted of preparing for a school day and traveling to school; ‘time at school’ included regular academic curricula, PE lessons, and recess time (including morning and afternoon recess); and ‘time after school’ included, for instance, traveling to home, outdoor plays, sports participation, performing homework, and screen time. 

Of the 189 children with low MC, 127 (67.2%) consented to wear an accelerometer, which was slightly higher than the whole sample of the SALTO study (62.6%), indicating no selective drop-out in the low MC group. Weight status was objectively assessed by a calibrated portable electronic scale (model 803; SECA, Hamburg, Germany) and length by a SECA portable stadiometer (model 213; SECA, Hamburg, Germany). BMI z-scores were calculated based on a Dutch reference population [41] and these scores were classified into four categories (underweight (BMI z-score ≤ −1.65), healthy weight (−1.64 ≤ BMI z-score ≤ 1.03), overweight (1.04 ≤ BMI z-score ≤ 1.64), and obese (BMI z-score ≥ 1.65)). Further, gender, grade, and age (date of birth) were measured.

### 2.3. Data Analysis

Analyses were performed with SPSS Statistics, version 24 (IBM Corp., Armonk, NY, USA). The level of significance was defined at *p* < 0.05 in all analyses. Daily MVPA and SB were analyzed both in absolute minutes per day and share of time per day. Additionally, share of time per day was analyzed for three different time intervals: time before school, time during school, and time after school. Differences in share of time spent in MVPA and SB for gender and age (operationalized as grade-4 and grade-6 children) were analyzed by Independent Sample T-tests. Moreover, differences in MVPA and SB (percentages) between time before school, time at school, and time after school were analyzed by ANOVAs with post hoc tests (Tukey).

## 3. Results

In total, 117 of 127 (92.1%) provided valid accelerometer data during all three time periods for at least three weekdays. On average, children provided 3.96 valid weekdays of accelerometer data. Total daily wear time was 784.8 min, of which 328.6 min per day (41.9%) were measured during school. No differences in wear time were found for gender or grade. On average the study sample was 9.18 years old, and about one in five children were overweight (Table 1). Boys and girls were equally represented, and grade-6 children were overrepresented in this sample.

On average children spent 50.7 (SD = 20.7) min per day in MVPA and one in four children engaged in at least 60 min of MVPA per day. Boys were significantly more active than girls (*p* < 0.01), both in absolute time and share of time spent in MVPA (57.6–43.6 min/day, 7.3%–5.6%, respectively). In addition, children spent 513.9 (SD = 55.7) min per day in SB and girls showed both higher absolute minutes and share of time per day in SB (524.8–503.2 min/day and 67.3%–63.8% respectively; Table 2). 

Grade-4 and grade-6 children showed comparable results on absolute time per day and share of time spent per day in MVPA. In contrast, for SB a significant difference was found between both groups. Grade-6 children showed significant higher levels of SB compared to grade-4 children (521.9 versus 489.7 min/day and 66.5% versus 62.6%, respectively), while no difference in wear time was noticed, i.e., the increase is not the result of prolonged waking hours of older children (Table 2). 

Throughout a weekday, children accumulated 8.3% of total daily time in MVPA before school, 37.3% during school, and 54.4% after school. For SB, a total of 8.6% of total daily sedentariness was performed before school, 44.1% during school, and 47.3% after school. Concerning segmented time periods, children showed relatively more MVPA before and after school (6.5% versus 7.1% min/day) compared to during school (5.5%; both *p* < 0.01). Moreover, children spent a significantly higher percentage of time sedentary during school (69.0%) compared to before (62.0%; *p* < 0.01) and after (63.3%; *p* < 0.01) school. A similar pattern for MVPA and SB was found in both genders separately, but the difference in time spent in MVPA between before and during school was not significant for both boys and girls, whereas the differences between during and after school were significant for both genders (*p* = 0.03 both for boys and girls). Gender-specific SB analyses revealed an equal pattern as nonstratified analyses. Both boys and girls showed significantly higher levels of SB during school compared to before and after school (*p* < 0.01).

Before school, boys showed significantly higher levels of MVPA than girls (7.3% versus 5.6%), whereas no differences were found in time spent sedentary. No differences were found in time spent in either MVPA or SB for children attending grade-4 or grade-6 (Table 2). 

During school, boys showed significantly higher levels of MVPA than girls. Though differences in time spent in MVPA, boys and girls spent an equal share of total daily MVPA during school (36.8%–37.8% of total daily MVPA). For SB, gender differences were found during school as well. Boys showed favorable outcomes compared to girls. During school, no differences in MVPA were found between grade-4 and grade-6 children, but a significant difference was found between both grades in share of time spent in SB. Grade-6 children were more sedentary during a school day than children attending grade-4 (Table 2).

After school, a significant difference was found between boys and girls for MVPA. Boys spent substantially more time in MVPA compared to girls. For SB, no gender differences were found. In addition, no differences in MVPA were found between grade-4 and grade-6 children after school, whereas grade-6 children showed higher levels of SB after school (Table 2).

## 4. Discussion

### 4.1. Main Findings

The purpose of the current study was to disentangle physical activity (PA) behavioral patterns in children with low motor competence (MC) at school days, while taking into account potential differences in gender and grade. The main finding of this study was that children with low MC are less active at school compared to other time periods during a school day, i.e., before and after school. Children spent both more time in SB and less time in MVPA during school. At school, children should spend approximately 20 min less in SB to equalize sedentary levels they accumulated during the before and after school period (23 min compared to before school and 19 min compared to after school, respectively). In addition, during all time periods at a school day, girls showed lower levels of PA compared to boys, which is in line with the literature [24,29,33,42]. Also, grade-6 children spent more time in SB compared to grade-4 children [23,26,43], while time spent in MVPA did not differ significantly. The increased levels of SB in grade-6 children might be partially explained by higher academic demands, both during school as well as after school (homework). As no changes in MVPA were found, higher levels of SB were likely derived from a shift from light PA to SB. Intervening on gradual decreases in PA turning in sedentary levels need to be considered to prevent age-related increases in SB and declines in PA. Finally, only 25% of children with low motor skills engaged in the PA guidelines of 60 min of MVPA per day, which is about half of the Dutch average [44]. De Meester et al. [11] found similar results indicating that children with low MC are less likely to meet PA guidelines than children with average or high MC. On average, children with low MC spent 51 min per school day in MVPA, which is lower than studies in the Netherlands and other European countries with similar age groups and methods to assess MVPA conducted in the same season [45,46,47]. These lower levels of MVPA might be explained by the inclusion of all children regardless their level of motor competence, i.e., children with normal or high MC, who most likely show higher levels of MVPA. This emphasized the need to focus on vulnerable or high-risk groups in particular, such as children with low MC. Further research should investigate which factors specifically predict activity levels of children with low MC in order to develop effective interventions to stimulate their PA levels. Within this high-risk population, i.e., children with low MC, the differences between boys and girls were comparable with other studies [48,49] and accounted for up to 25% less time spent in MVPA by girls. This substantial lack of MVPA in girls emphasizes the need to develop PA stimulating interventions for young girls in order to help them to develop healthy PA behavioral patterns during childhood. Several studies [3,13,16,17,50] suggested a reciprocal relationship between children’s MC and their PA and SB. In line with this, intervening on girls’ MC, for instance in PE lessons, holds potential to effectively affect girls’ MVPA levels [14]. However, in the current study no gender differences were found on MC (equal group distribution), but it should be noted that MC classification was based on age- and gender-specific cut-off points [37]. This strategy enables benchmarking and comparison of study results with other studies applying this protocol, but meanwhile masks the lower motor development in girls compared to boys in absolute terms as girls’ time to complete the track was significantly higher than boys. Beyond this focus on school hours, gender differences in MVPA found in the after school period emphasizes the need to focus on the broader system of PA, especially for girls, Like in any other period, girls spent 25% less time in MVPA (8 min) after school compared to boys. A possible explanation for this gender difference might partly be explained by object control skills. Boys generally show better performances in object skill control compared to girls [51] and object control is associated with higher levels of MVPA [52]. In addition, according to the moderating role of gender as proposed in the EnRG framework [21], environmental influences on PA might differ for boys and girls. A forthcoming implication is to study suitability and attractiveness of outdoor play and sports opportunities for girls. Future research on girls’ PA engagement is warranted.

Results of the current study indicate that schools are relatively the strongest contributor for the total time children with low MC spent in SB. Though school’s focus is primarily on cognitive tasks, school’s high level of contribution to inactivity is remarkable as multiple PA supportive domains, i.e., PE lessons [29,33] and recess [30,49], were included in this time period. Additionally, taking into account the potential reach schools, the school should be considered as a suitable and best setting to promote PA in children with low MC, e.g., by considering well-designed curriculum-based PA or additional time scheduled for PA activities, particularly suitable for girls. By contrast, a study by Beck and colleagues found that children were more sedentary during out of school hours than during school hours [34]. Differences might be explained by the focus on low MC-children in our study, whereas Beck’s study made no distinction in children’s motor development. Furthermore, (socio-cultural) differences in the U.S. and Dutch school systems may have contributed to the different findings.

### 4.2. Strengths and Limitations

A study strength was the segmentation of school days in meaningful time periods to identify potential (time) periods to effectively affect PA, SB, and motor skill development. Another strength of this study is that PA was objectively measured and that the compliance of the participants was high, i.e., the average mean accelerometer wear time was over 13 h per day during waking hours for four school days. A limitation of this study was the overrepresentation of grade-6 children with low MC compared to grade-4 children, while in the whole research population, the number of children in both groups was equally distributed [12]. Also, the study only focused on weekday patterns. Weekend day patterns were considered to more heterogeneous in terms of PA behavioral patterns, limiting the opportunity to define meaningful periods to intervene for larger populations. Further, as the purpose of the study was to increase insight in PA and SB behavioral patterns in children with low MC, only children with low MC were included and no comparison to other MC groups (normal or highly competent) could be made. Additionally, the use of the Athletic Skills Track (AST) could have influenced the clustering of children into low or very low motor development. The AST is a circuit-based assessment tool and not a product- or process-assessment tool, which is developed to be useful and feasible to conduct within PE lessons. Despite a quite strong correlation between the AST and more often used instruments, such as Körperkoördinationstest für Kinder (KTK) [53], using the AST does not allow to differentiate between types of poor motor ability (e.g., locomotor or stability skills and/or specific motor activities, such as running and clambering). Moreover, in the current study the term motor competence was used as an outcome for the AST. Yet, it should be noticed that this outcome measure only assessed a small part of the broad spectrum covered by the ‘umbrella term’ motor competence, which impacts comparability with other studies using more extensive measures to assess MC [3,6,54]. Further, using the AST may have impacted the classification of children in the category low MC, and we cannot rule out any misclassification caused by unmeasured concepts of MC. Finally, the cross-sectional design of the current study does not allow to draw any conclusions on causality and reciprocity between MC and PA levels.

## 5. Conclusions

Time spent at school is relatively the strongest contributor to sedentary behavior in children with low motor competence. The negative impact of school hours on children’s SB seems to be higher in girls compared to boys. Therefore, time spent at school may also be considered as the most promising period to reduce SB and stimulate PA in children with low MC. High-risk groups, either in terms of MC, gender, or age show deviant PA levels and should be better targeted in (school-based) PA supportive interventions by optimally fitting PA interventions to their needs, rather than apply generic interventions.

## Figures and Tables

**Table 1:** Descriptive statistics for total population and stratified for gender and grade.

	Total (*N* = 117)	Girls (*N* = 58)	Boys (*N* = 59)	Grade-4 (*N* = 29)	Grade-6 (*N* = 88)
**Demographics**					
Age (years)	9.18	9.12	9.24	7.86	9.61
Length (cm)	140.18 ± 8.64	139.34 ± 8.73	141.01 ± 8.55	131.68 ± 6.31	143.05 ± 7.35
Weight (kg)	34.22 ± 8.53	34.15 ± 7.79	34.29 ± 9.28	28.19 ± 4.41	36.25 ± 8.64
Overweight (%)	20.9%	22.8%	19.0%	13.8%	22.7%
**Wear time (min ± SD)**					
Daily	784.79 ± 52.57	779.33 ± 50.13	790.16 ± 54.76	783.14 ± 50.58	785.33 ± 53.49
Before school	70.36 ± 22.26	68.94 ± 21.62	71.75 ± 22.98	77.91 ± 27.59	67.87 ± 19.77
During school	328.78 ± 18.07	327.95 ± 18.12	329.60 ± 18.15	332.79 ± 19.18	327.46 ± 17.61
After school	385.65 ± 54.66	382.43 ± 52.79	388.81 ± 56.72	372.44 ± 56.51	390.00 ± 53.66

**Table 2:** Moderate-to-vigorous physical activity (MVPA) and sedentary behavior (SB) segmented for time periods and stratified by gender and age.

PA Levels	Total (*N* = 117)	Girls (*N* = 58)	Boys (*N* = 59)	*p* (t)	Grade-4 (*N* = 29)	Grade-6 (*N* = 88)	*p* (t)
**Non-segmented school day**							
Total daily MVPA (min)	50.65 ± 20.67	43.56 ± 15.33	57.62 ± 22.88	<0.01 (−3.911)	51.63 ± 17.54	50.33 ± 21.68	0.77 (0.294)
% in MVPA per day	6.43 ± 2.56	5.58 ± 1.93	7.26 ± 2.83	<0.01 (−3.761)	6.58 ± 2.19	6.38 ± 2.67	0.71 (0.370)
Total daily SB (min)	513.93 ± 55.67	524.84 ± 57.37	503.21 ± 52.22	0.04 (2.133)	489.67 ± 51.41	521.93 ± 54.95	<0.01 (−2.784)
% in SB per day	65.56 ± 6.30	67.32 ± 5.56	63.82 ± 6.55	<0.01 (3.112)	62.58 ± 5.76	66.54 ± 6.19	<0.01 (−3.039)
**Before school**							
% in MVPA per segment	6.45 ± 3.79	5.68 ± 3.24	7.21 ± 4.15	0.03 (−2.216)	5.75 ± 2.75	6.68 ± 4.06	0.17 (−1.401)
% in SB per segment	62.04 ± 8.66	62.61 ± 8.67	61.48 ± 8.69	0.48 (0.702)	63.61 ± 9.54	61.52 ± 8.35	0.26 (1.126)
**At school**							
% in MVPA per segment	5.50 ± 2.52	4.78 ± 1.87	6.22 ± 2.86	<0.01 (−3.227)	5.61 ± 3.05	5.47 ± 2.33	0.79 (0.267)
% in SB per segment	68.95 ± 6.59	71.19 ± 5.35	66.75 ± 6.99	<0.01 (3.852)	66.30 ± 6.81	69.82 ± 6.32	0.01 (−2.552)
**After school**							
% in MVPA per segment	7.05 ± 3.61	6.14 ± 3.11	7.94 ± 3.87	<0.01 (−2.765)	7.71 ± 3.02	6.83 ± 3.78	0.25 (1.151)
% in SB per segment	63.28 ± 8.78	64.73 ± 7.97	61.86 ± 9.37	0.08 (1.783)	58.87 ± 7.46	64.73 ± 8.74	<0.01 (−3.244)
